# Suppressing Polarization Mode Dispersion with the Quantum Zeno Effect

**DOI:** 10.3390/e27010027

**Published:** 2025-01-01

**Authors:** Ian Nodurft, Alejandro Rodriguez Perez, Naveed Naimipour, Harry C. Shaw

**Affiliations:** 1 Peraton, Reston, VA 20190, USA; inodurft@peraton.com; 2 NASA Goddard Space Flight Center, 8800 Greenbelt Rd., Greenbelt, MD 20771, USA; alejandro.rodriguezperez@nasa.gov (A.R.P.); naveed.naimipour@nasa.gov (N.N.)

**Keywords:** Zeno, polarization mode dispersion, polarization, quantum Zeno effect, PMD, optical fibers, fibers, waveguides

## Abstract

Polarization mode dispersion can introduce quantum decoherence in polarization encoded information, limiting the range of quantum communications protocols. Therefore, strategies to nullify the effect would reduce quantum decoherence and potentially increase the operational range of such technology. We constructed a quantum model of polarization mode dispersion alongside a two-level absorbing material. The two-level material serves to destructively measure one of two orthogonal polarization modes, thus projecting the polarization onto the other state. The theoretical results are supported by a numerical simulation in Mathematica Documentation where we compare the evolution of the polarization state with and without the absorbing material. We find that this strategy is effective in suppressing the effects of polarization mode dispersion, and that this method produces a global phase shift related to the waveguide’s birefringent properties.

## 1. Introduction

Polarization mode dispersion (PMD) is a near unavoidable feature of fiber optical communications, wherein the polarization state of light passing through a medium (optical fibers) shifts as it travels [1]. In fibers, for example, this is a consequence of birefringence present in the waveguide due to unintentional variations in the shape of the core [1,2,3,4]. Various environmental effects (wind, temperature, etc.) can cause the fibers to bend and twist, further randomizing the birefringence and introducing PMD into the fiber [5]. Strategies employed to compensate for and avoid it include intentionally exaggerating the birefringence such that cross-talk between two orthogonal polarization states is negligible, creating polarization-maintaining fibers (PMFs) [1,6,7,8,9], among other methods [10,11]. Unfortunately, due to the significant delay in one polarization with respect to the other, the large birefringence of PMFs induces decoherence in entangled polarization states.

PMD can be particularly detrimental to quantum communications as qubit-encoded (especially entangled) states in the polarization degree of freedom are susceptible to decoherence or errors as a result, limiting the range of quantum communications relying on such states. Some errors can be avoided or corrected through the use of cluster states [12,13,14] and error-correcting codes [15], but these, of course, have their limitations such as scalability issues from large overhead in required physical resources or that they cannot be reversed. Quantum repeaters [16] are another clear method to circumvent these issues, though they of course rely on quantum teleportation, itself a communication protocol, and as such are subject to the same limitations. In other words, improving the distance over which quantum coherence is maintained inherently improves the utility of repeaters.

Our proposed method relies instead on the quantum Zeno effect, the phenomenon in which repeated measurements (or interactions) prevent a quantum system from evolving [17,18]. Creative use of the Zeno effect can induce unusual phase shifts or turn probabilistic processes into fully deterministic ones and it has been proposed for several such purposes [19,20,21,22], and experimentally demonstrated [23].

In our case, the Zeno effect is induced by the introduction of a two-level absorbing medium into the evanescent field of an optical waveguide (fiber for instance). The absorbing medium will be assumed on resonance with a given frequency of either right- or left-circular polarized photons, while the photon passing through will be the opposite polarization. Such dipole transitions are known to follow selection rules based on the angular momentum difference $\Delta m_{l}$ between the ground and excited states [24]. In this case, absorption (emission) of left-circular polarization changes the absorber’s quantum number $m_{l}$ by −1 (+1) while right-circular polarization has the inverse relationship. Materials with these properties, such as rubidium vapor, are common in experiments that require optical pumping or electromagnetically induced transparency [25,26,27].

**Figure 1:**
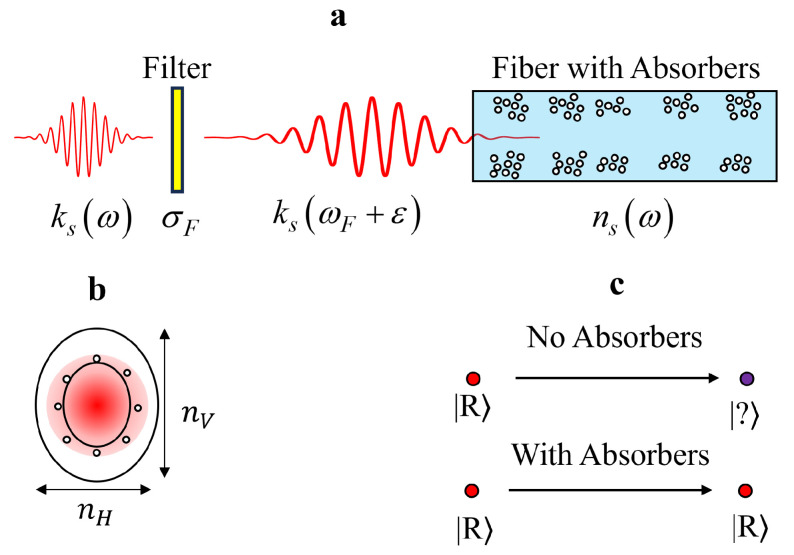
Visual outline of the proposed PMD suppression technique. (**a**) Outline of major assumptions of the scenario, with a single photon passing through a narrow-bandwidth filter, followed by entering an optical waveguide (fiber) containing an absorbing medium. (**b**) An example cross-section of an optical fiber overlaid with an electromagnetic field, with an elliptical shape rather than the ideal circular shape. This causes the differential refractive index between horizontal and vertical polarizations. (**c**) A simple depiction of the polarization state at the input and output of a fiber with and without the absorbing medium. Without absorbers, the polarization is unknown at the end, whereas the polarization is preserved with the absorbers present.

Without loss of generality, we will assume throughout this paper that the absorbing medium is resonant with left-circularly polarized photons while the photons traveling through the fiber will be assumed right-circularly polarized. The presence of the absorbing material will serve to destroy any left-circularly polarized photons that appear in the waveguide, while letting right-circular photons through unhindered. The proposed design and its operation are depicted in Figure 1. By suppressing the effects of birefringence, it is in principle possible to transmit arbitrary qubits encoded in the polarization degree of freedom without introducing any decoherence.

In Section 2, we will construct a theoretical model for PMD acting on a single photon of right-circular polarization, followed by a derivation of the Zeno effect acting on the photon in such an environment. Section 3 will show the results of a numerical simulation corroborating the theoretical predictions, Section 4 will briefly overview how this scheme might be implemented, and we will conclude this paper in Section 5.

## 2. Theory

We consider the situation depicted in Figure 1. A single photon is injected into an optical fiber after passing through a narrow frequency-bandwidth filter. The general single-photon state is given by [28,29]

(1) $$|\psi \left(0\right)\rangle =c_{N}\int d\omega g\left(\omega \right){\hat{a}}_{k,s}^{†}|0\rangle .$$

The function g(ω) is determined by the source. Introducing a narrow-bandwidth filter replaces g(ω) with a Gaussian distribution centered on frequency $\omega_{F}$. The constant $c_{N}$ is a suitable normalization constant, and the subscripts *k* and *s* refer to the wavenumber and polarization, respectively. To obtain the time-dependence of the polarization, we apply the positive-frequency component of the electric field operator in one dimension [30]:

(2) $$\hat{E}\left(x,t\right)=i\sum_{k,s}\sqrt{\frac{2\pi \hbar \omega_{k}}{V}}{\hat{e}}_{k,s}e^{i(kx-\omega_{k}t)}{\hat{a}}_{k,s}.$$

Applying Equation (2) to Equation (1), and replacing $g\left(\omega \right)\to \exp\left[-{\left(\omega -\omega_{F}\right)}^{2}/\sigma_{F}^{2}\right]$, we obtain the time-dependent state

(3) $$|\psi \left(t\right)\rangle =c_{N}^{\prime }\int d\omega \sqrt{\omega }e^{\frac{-{\left(\omega -\omega_{F}\right)}^{2}}{\sigma_{F}^{2}}}e^{-i\omega t}[e^{ik_{H}x_{H}}|H\rangle -ie^{ik_{V}x_{V}}\left|V\rangle \right]$$

where we have further assumed that the photon begins in the polarization state $|R\rangle =(|H\rangle -i|V\rangle )/\sqrt{2}$.

Note that the wavenumber is different for the two polarization states as a consequence of the birefringent medium. In general, these values will themselves change with time, but we have assumed a constant birefringence. This is valid provided that the interaction strength between the absorbing medium and light is strong compared to the degree of birefringence.

We will leave the majority of this derivation for Appendix A.1, but a few important replacements will be noted here. We first assume constant birefringence, and the narrow-bandwidth filter permits us to perform a Taylor expansion of the wavenumber about the filter frequency

(4) $$k_{s}=k_{0,s}+\alpha_{s}\left(\omega -\omega_{F}\right)+\beta_{s}{\left(\omega -\omega_{F}\right)}^{2}$$

and the frequency under the integral is replaced by

(5) $$\omega =\omega_{F}+\epsilon .$$

As noted in Appendix A.1, the narrow-bandwidth filter permits the replacement of $\sqrt{\omega }$ with a constant value $\sqrt{\omega_{F}}$, simplifying the integrand, after which the integral is taken over by all frequencies as an approximation. Physically, the narrow-bandwidth filter limits the range of frequencies present in the fiber such that higher-order effects of the refractive index other than dispersion $\beta_{s}$ are negligible. We also replace $x_{s}\to v_{s}t=ct/n_{s}$, where $n_{s}$ is the refractive index of an *s* polarized photon.

Evaluating the integral (3) under these approximations, we obtain

(6) $$|\psi \left(t\right)\rangle =c_{N}^{\prime \prime }\left(\frac{e^{\frac{{\left(\alpha_{H}x_{H}-t\right)}^{2}}{4(-\frac{1}{2\sigma_{F}^{2}}+i\beta_{H}x_{H})}}}{\sqrt{-\frac{1}{2\sigma_{F}^{2}}+i\beta_{H}x_{H}}}|H\rangle -i\frac{e^{\frac{{\left(\alpha_{V}x_{V}-t\right)}^{2}}{4(-\frac{1}{2\sigma_{F}^{2}}+i\beta_{V}x_{V})}}}{\sqrt{-\frac{1}{2\sigma_{F}^{2}}+i\beta_{V}x_{V}}}|V\rangle \right).$$

This general result is reducible to

(7) $$|\psi \left(t\right)\rangle \approx \frac{1}{\sqrt{2}}\left(e^{-i\phi_{H}t}|H\rangle -ie^{-i\phi_{V}t}|V\rangle \right)$$

where

(8) $$\phi_{s}=\left\{\begin{array}{cc} 2\sigma_{F}^{2}c\frac{\beta_{s}}{n_{s}}, & t\to 0^{+}\\ \frac{n_{s}}{4\beta_{s}c}{\left(\frac{\alpha_{s}}{n_{s}}-1\right)}^{2}, & t\to \infty \end{array}\right..$$

Note that the convenient form of the polarization time-dependence in (7) is valid for timescales much smaller or larger than $t=n_{s}/\left(2\sigma_{F}^{2}|\beta_{s}|c\right)$ for either polarization *s*.

A time-evolution operator is easily obtained by inspection, from (7),

(9) $$\hat{U}\left(t\right)=\left[\begin{array}{cc} e^{-i\phi_{H}t} & 0\\ 0 & e^{-i\phi_{V}t} \end{array}\right],$$

from which we can derive the dynamics of the system under continuous observation [18]. Projecting this operator an infinite number of times within a finite time *T* produces the effective time-evolution operator:

(10) $$\hat{V}\left(T\right)=e^{\frac{i}{2}\left(\phi_{H}+\phi_{V}\right)T}\left|R\rangle \langle R\right|$$

The full derivation is provided in Appendix A.2. Interestingly, the phase shift of the polarized state under the Zeno effect is equivalent to the average of the horizontal and vertical phase shifts, necessarily those of the short timescale in (8).

The question remains, what conditions are required for the Zeno effect to be successful? In general, the physical situation in which the Zeno effect arises is any of those cases where the transition into some undesired state (in our case, the opposite polarization) is much slower than the higher-order transition into and then out of the undesired state into another “measuring” state (in our case, an excited absorber state). In such a situation, any growth in the population of the undesired state is quickly lost to the measuring state, ultimately leaving the undesired state at 0 probability.

To this end, we calculate the relevant transition rates for |R〉|G〉→|L〉|G〉 and |R〉|G〉→|R〉|E〉. The first- and second-order transition probabilities as a function of time are calculated using the formulae [31]

(11) $$P_{i\to f}\left(t\right)=|\frac{1}{i\hbar }\int_{t_{i}}^{t_{f}}dt^{\prime }\langle f|{\hat{H}}_{I}\left(t^{\prime }\right)|i\rangle {|}^{2}$$

and

(12) $$P_{i\to f}\left(t\right)=|\frac{1}{{\left(i\hbar \right)}^{2}}\int_{t_{i}}^{t_{f}}dt^{\prime }\int_{t_{i}}^{t_{f}}dt^{\prime \prime }\sum_{m}\langle f|{\hat{H}}_{I}\left(t^{\prime }\right)\left|m\rangle \langle m\right|{\hat{H}}_{I}\left(t^{\prime \prime }\right)|i\rangle {|}^{2}.$$

Here, ${\hat{H}}_{I}\left(t\right)$ is the time-dependent interaction Hamiltonian and *i* and *f* denote the initial and final states of the system, respectively. The Hamiltonian of our system is given by

(13) $$\hat{H}={\hat{H}}_{0}+{\hat{H}}_{I}$$

where

(14) $${\hat{H}}_{0}=\hbar \omega \left({\hat{a}}_{R}^{†}{\hat{a}}_{R}+{\hat{a}}_{L}^{†}{\hat{a}}_{L}+1\right)+\frac{1}{2}\omega_{0}{\hat{\sigma }}_{z}$$

is the rest Hamiltonian and

(15) $${\hat{H}}_{I}=\hbar \hat{\Phi }+\hbar \lambda \left({\hat{a}}_{L}{\hat{\sigma }}^{+}+{\hat{a}}_{L}^{†}{\hat{\sigma }}^{-}\right)$$

is the interaction Hamiltonian. Finally, ω is the frequency of light and $\omega_{0}$ is the resonant frequency of the two-level absorber, and these are assumed equal in the calculation. The parameter λ is the coupling between the absorber and the light, and $\sigma_{z}$ is the Pauli Z spin operator with ${\hat{\sigma }}^{\pm }$ the Pauli ladder operators, given in matrix form as

(16) $${\hat{\sigma }}^{+}=\left[\begin{array}{cc} 0 & 1\\ 0 & 0 \end{array}\right],\;{\hat{\sigma }}^{-}=\left[\begin{array}{cc} 0 & 0\\ 1 & 0 \end{array}\right],\;{\hat{\sigma }}_{z}=\left[\begin{array}{cc} 1 & 0\\ 0 & -1 \end{array}\right]$$

These operators are those used in the the Jaynes–Cummings model, wherein ${\hat{\sigma }}^{+}$ facilitates transitions out of the ground state |G〉 and into the excited state |E〉 while ${\hat{\sigma }}^{-}$ facilitates the reverse transition. ${\hat{\sigma }}_{z}$ returns the energy of the ground and excited states [30].

The phase component of the interaction Hamiltonian, $\hat{\Phi }$, is obtained from Equation (9) and is given by

(17) $$\hat{\Phi }=\left[\begin{array}{cc} \phi_{H} & 0\\ 0 & \phi_{V} \end{array}\right]$$

in the linear basis. $\hat{\Phi }$ facilitates the phase shift in the horizontal and vertical components of the polarization due to PMD. In evaluating (11) and (12), our initial and final states are in terms of circular polarization states, but we perform the calculation in the linear basis, rewriting ${\hat{a}}_{R}^{†}\to \left({\hat{a}}_{H}^{†}-i{\hat{a}}_{V}^{†}\right)/\sqrt{2}$ and ${\hat{a}}_{R}^{†}\to \left({\hat{a}}_{H}^{†}+i{\hat{a}}_{V}^{†}\right)/\sqrt{2}$ and their corresponding lowering operators.

With all this, we calculate the first- and second-order transitions from an initial state |i〉=|R〉|G〉, into final states |f〉=|L〉|G〉 and |f〉=|0〉|E〉. Our time-dependent transition probabilities, $P_{RG\to LG}$ and $P_{RG\to 0G}$, are therefore

(18) $$P_{RG\to LG}\left(t\right)=\frac{t^{2}}{4}{\left(\phi_{H}-\phi_{V}\right)}^{2}+\frac{t^{4}}{16}{\left(\phi_{H}^{2}-\phi_{V}^{2}\right)}^{2}$$

and

(19) $$P_{RG\to 0E}\left(t\right)=\frac{\lambda^{2}t^{4}}{32}{\left(\phi_{H}-\phi_{V}\right)}^{2}.$$

Note that the result in (18) has both a first- and second-order contribution while that of (19) only has a second-order contribution as the first-order probability is 0. Similar results can be obtained for the opposite case (left-polarized photon into right-polarized and excited state of a right absorber).

Taking the ratio of the probabilities, $P_{\phi /E}=P_{RG\to LG}/P_{RG\to 0E}$, provides some insight into the necessary conditions for the Zeno effect. Conditions under which the ratio is small indicate that the absorption rate is much larger than the transition rate to left-circular polarization. The ratio as a function of time is

(20) $$P_{\phi /E}\left(t\right)=\frac{8}{\lambda^{2}t^{2}}+\frac{2}{\lambda^{2}}{\left(\phi_{H}+\phi_{V}\right)}^{2}.$$

As long as the coupling parameter between the absorbing medium and the photon mode, λ, is significantly larger than the sum of the horizontal and vertical phase shift parameters, then the second term disappears. The same cannot be said for the first term, where the inverse dependence on time means that there is always some short time period during which the phase transition is faster than the absorption. Thankfully, this can be mitigated by increasing the coupling parameter and consequently decreasing the time during which PMD may proceed. In a physical sense, this would be accomplished by either placing the absorbing medium deeper within the evanescent field or by simply increasing the density of the absorbing medium around the field. Either method introduces an effective increase in absorption rate. Nevertheless, the ratio is only large when $t∼\lambda^{-1}$ or smaller, after which the ratio declines, meaning that as long as λ is sufficiently large, the time over which the polarization can shift will be quite small, meaning the accumulated probability that the photon will shift in polarization will remain small.

## 3. Numerical Simulation

In the previous section, we looked at the theoretical case of a photon passing through a waveguide of constant birefringence, determined the time-dependence of the polarization, and constructed an approximate time-evolution operator producing PMD. From here, we were able to determine an effective time-evolution operator from a Zeno effect, and determine transition probabilities of polarization shift and absorption as a function of time, providing some insight into necessary conditions for a Zeno effect.

**Figure 2:**
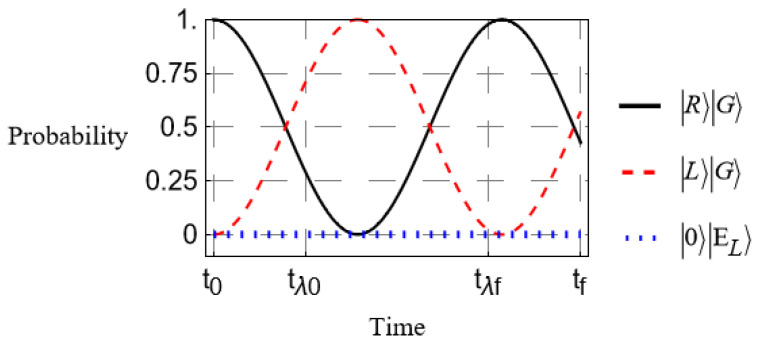
Time-evolution of the polarization state in the approximate form given in Equation (7) as it propagates through a birefringent medium. No absorbers are present. The black curve is the probability that the photon is right-polarized, the red dashed curve signifies left polarization, and the blue dotted curve represents the probability of excitation of the absorbers.

In this section, we perform a numerical simulation in Mathematica Documentation of our physical system using the model derived in the previous section. We look at a few relevant cases where we vary the time-dependence of the absorber coupling and the birefringence. Each case is compared with the evolution in the absence of absorbers, shown in Figure 2. The time-dependence from the short-time regime of Equation (8) is shown here.

The simulation is performed by first constructing the Hamiltonian followed by solving the time-dependent Schrodinger equation [32]:

(21) $$i\hbar \frac{d|\psi (t)\rangle }{dt}=\hat{H}\left(t\right)|\psi \left(t\right)\rangle .$$

**Figure 3:**
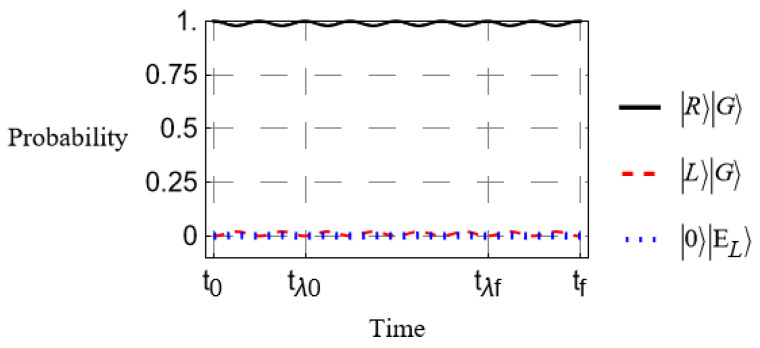
Time-evolution of the polarization state in the presence of absorbers. All curves are labeled as in Figure 2.

We assume that the initial state is $|\psi (t_{0})\rangle =|R\rangle |G\rangle$ and $\hat{H}\left(t\right)$ is taken from Equations (13)–(15). We further assume an infinite number of absorbers with which the incident photon interacts as it travels through the birefringent medium; therefore, the coupling parameters are approximated as a single, continuous potential. In our first case, we examine the case where, as in our theoretical treatment in the last section, the birefringence and coupling to the absorbers is held constant. That is, the $\phi_{s}$ does not vary in time, nor does the coupling parameter, λ. This simplest case is shown in Figure 3.

We see from Figure 2 that in the absence of absorbers, the polarization shifts cyclically as expected. Comparing with Figure 3, we see that introducing the absorbing medium drastically reduces the probability of transition at any time. Increasing the coupling parameter λ continues this trend to make transitions negligible in probability, as expected, thus realizing the total suppression of PMD. As predicted in the previous section, the required coupling strength for an effective PMD suppression increases along with the degree of birefringence. In the example of Figure 3, the difference $\phi_{H}-\phi_{V}$ is only about a 1% difference from their average.

**Figure 4:**
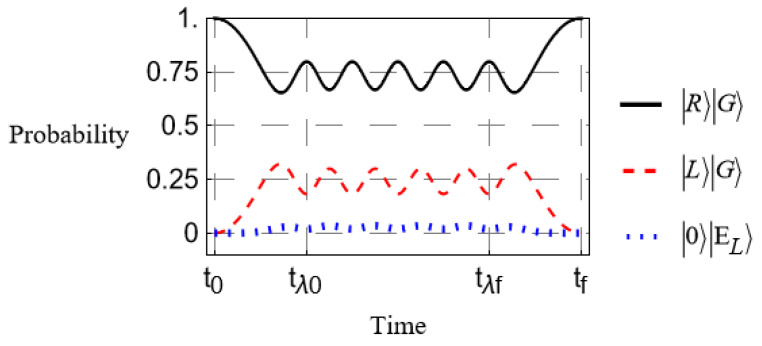
Time-evolution under an adiabatic approximation. The coupling parameter λ is slowly turned on until it reaches a maximum at time $t_{\lambda 0}$ and is slowly turned off at time $t_{\lambda f}$.

**Figure 5:**
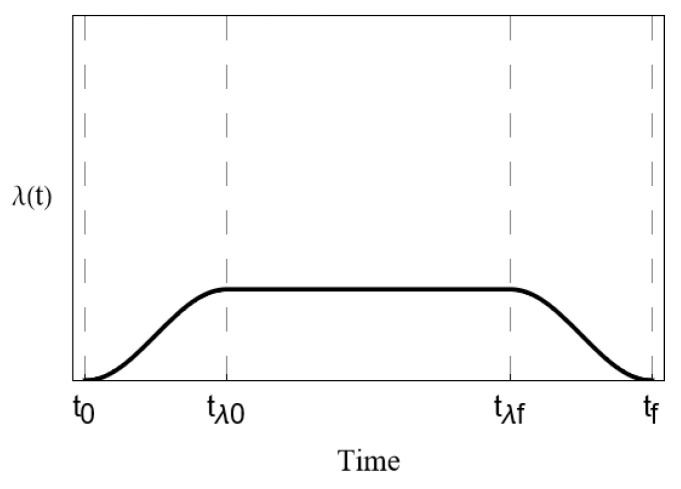
Time-evolution of the coupling parameter λ(t) assuming an adiabatic (slowly turned on) potential.

As a matter of interest, we compare an additional scenario. Shown in Figure 4 is a simulation in which we assume an adiabatic approximation, where we slowly turn on the absorber coupling [31]. This case may be somewhat more applicable to a physical implementation of this scheme, as it will generally be the case that PMD will begin to affect an incident photon before it has a chance to interact with any additional absorbing material within the waveguide’s path. The time-dependence of λ(t) is shown in Figure 5.

**Figure 6:**
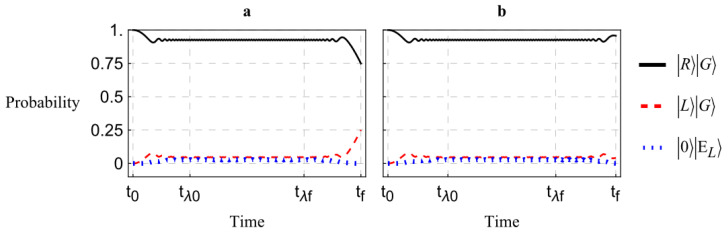
Time-evolution under the assumption that the coupling parameter λ is turned on slowly. λ is chosen to be especially strong for a significant suppression of PMD. Both plots are the same, but in (**a**), $t_{\lambda f}-t_{\lambda 0}$ is arbitrarily larger than that same period in (**b**).

We have made the same choice for the coupling parameter in Figure 4 as in Figure 3. Notice that while the probability remains significantly reduced, it is not nearly as good as in Figure 3. Increasing the coupling reduces this just as in the other case, but it requires much stronger coupling to reach similar suppression. Furthermore, the adiabatic “turn off” of the coupling can lead to inconsistent outcomes. See Figure 6 for a comparison between two somewhat different lengths of time over which the coupling parameter is at a maximum. The coupling parameter here is 5 times larger than in the previous examples, so suppression is fairly strong overall, but simply shortening the maximum coupling time $t_{f}-t_{0}$ by an arbitrary choice of 10% (5% in the total time of the simulation) yields a much smaller probability that the photon exits in a left-polarized state. The case of Figure 4 ending in a nearly perfect right-polarized state is nothing more than a happy coincidence. It is uncertain what exactly the minimum transition rate can be for any given coupling parameter, “turn on” rate, etc. though in the case of Figure 6, we found probabilities as low as 59.6% remaining in the right-polarized state.

This is due to the significant transition rate in the short time regime, as expected from the analysis of Equation (20). This does not, however, necessarily pose an issue if the coupling is suddenly “turned off”, as would be the case when the photon exits the waveguide, for instance. Whether this would introduce any quantum decoherence from which-path information [33] is as of yet unclear. Ultimately, this suggests that for implementations of this design, it is of great importance that the incident photon experiences strong interaction with the absorbing medium quite early, or else the output will become much harder to control.

These numerical simulations demonstrate, as a proof of concept, that the Zeno effect can be used to suppress the polarization mode dispersion under appropriate conditions [27].

## 4. Physical Implementation Discussion

**Figure 7:**
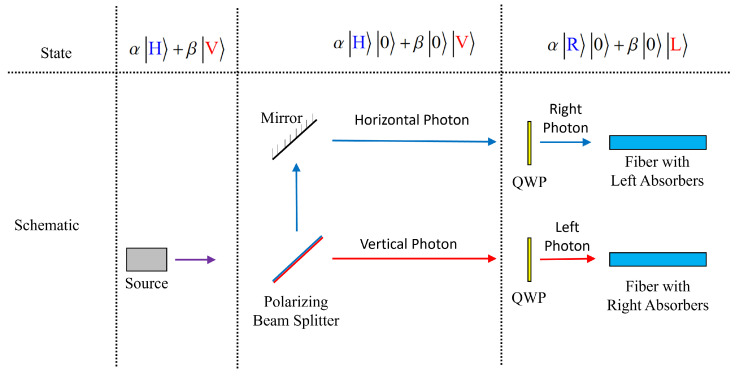
A schematic demonstrating an arbitrary polarization-encoded qubit protected via our proposed technique. A photon with arbitrary polarization is split into two paths by a polarizing beam splitter, followed by passing through quarter waveplates (QWPs) such that horizontal photons become right-polarized and vertical photons become left-polarized. The photon is split into two paths, with both paths protected by appropriate absorbers as they pass through optical fibers.

In this section, we will discuss how this technique might be used to protect the quantum coherence of an arbitrary qubit. Figure 7 shows a simple schematic depicting a photon of arbitray polarization |ψ〉=α|H〉+β|V〉, representing an arbitrary qubit where we could assign the logical representation |H〉=|0〉 and |V〉=|1〉. By splitting the photon into two paths with definite polarization with a polarizing beam splitter (PBS), the photon becomes entangled with the vacuum of the other mode, becoming

(22) $$\left|\psi \rangle \to \right|\psi^{\prime }\rangle =\alpha |H\rangle |0\rangle +\beta |0\rangle |V\rangle .$$

The spatially separated photon can be assigned the logical representation |H〉|0〉=|0〉 and |0〉|V〉=|1〉, in a 1-to-1 correspondence with the previous encoding. Finally, in spite of the fact that the polarization is not determined, it is known which path corresponds to a horizontal and a vertical photon, so it is a simple matter to place quarter waveplates (QWPs) in either path to convert |H〉→|R〉 and |V〉→|L〉.

At this stage, the quantum coherence of the system is entirely intact, though it has been transferred to a larger Hilbert space. Regardless, the photon then passes through a fiber with absorbers of right-circularly polarized photons or left-circularly polarized photons, preserving the polarization in either path, without determining the spatial path, and simultaneously the polarization, of the photon. After exiting the fiber, the photon may be recombined into one path with another pair of waveplates and a PBS, or simply measured depending on need. In either case, the quantum coherence of the qubit should remain preserved throughout the process, even if the physical qubit itself has been separated spatially.

The obvious cost in this situation comes from requiring a number of resources (fibers, waveplates) proportional to twice the number of qubits required for any given protocol. However, this may be an acceptable trade-off in situations where external environmental influences on waveguide birefringence are unavoidable.

Phase differences from environmental factors can be monitored and adjusted using the Hong–Ou–Mandel (HOM) interference, by adjusting the path length of one of the two paths to continually find the HOM dip for a photon pair with tight time correlation. For more details, see [34]. Other phase stabilization protocols are known. For instance, Ref. [35] employs a classical probe laser in a Mach–Zehnder configuration, actively measuring these fluctuations and correcting for them in real time.

Another important consideration is, what sort of materials might accomplish our goal? That is, absorb one circular polarization and not the other. Rubidium is commonly used, as an element with transitions that can absorb both left- and right-circular photons [27,36], with a 780 nm absorption line absorbing/emitting right-circular polarization and a 794.7 nm absorption line absorbing/emitting left-circular polarization. Quantum dots can be constructed with the absorption of circular polarization in mind [37], and thus, may be usable for our purposes as well with careful engineering.

It is worth emphasizing that a material which is close to resonance with a particular frequency of right- but not left-circularly polarized photons will not be the same as a material close to resonance with the same frequency of left- but not right-circularly polarized photons. Thus, two different materials roughly close to resonance with the desired frequency may be necessary. Other methods, such as the Stark or Zeeman effects, may be employed to manipulate quantum levels, increasing the available options for materials. Ultimately, employing this method on arbitrary polarization qubits will require careful consideration of spectroscopic properties to find an appropriate pairing.

## 5. Conclusions

Our investigation demonstrates that the quantum Zeno effect is a promising method for suppressing PMD in optical and quantum communications. It avoids probabilistic and scalability issues with cluster states and error-correcting codes [12,13,15] and allows for the preservation of arbitrary polarization states, but without the destruction of the quantum coherence of entangled polarization states that is present in PMFs. This last advantage, however, does require that the state be split into two separate waveguides as the technique will absorb one or the other circular polarization state as it passes through one waveguide since one of the two will be absorbed. Any quantum communication protocols relying on this technique, therefore, will necessarily require twice as many waveguides (fibers) as qubits.

Although other methods can promise similar results in the absence of atmospheric effects and temperature fluctuations, such environmental effects on the waveguides can also be mitigated with this proposed Zeno effect. In principle, such a technique would further avoid leaking which-path information to an environment, so that quantum coherence of entanglement is not destroyed. This is a problem in typical fiber communications in no small part due to the fact that the unknown stochastic nature of birefringence is exacerbated by atmospheric effects, causing the information to be rendered unusable for quantum protocols [5]. The technique described here would bypass these issues in either situation, though it is limited by the extent to which the phase shift in Equation (10) can be made equal. In a physical scenario, the absorbers of right-polarized photons may not be the same type, nor capable of reaching the same coupling or density as the absorbers of left-polarized photons. This could lead to somewhat different phase shifts through the fiber medium, and could affect the entanglement quality. Even so, bringing the phase shifts close enough together should extend the range over which entangled photons can be transmitted, most particularly if applied in regions of significant PMD. If the phase shifts are known, it can also be compensated for by simply adding phase delays.

In addition, the technique has potential for applications of nonlocal dispersion cancellation [28,29], conditioned on the proper engineering of the refractive indices. Indeed, nonlocal cancellation of dispersion may be simply combined with this technique to further improve the transmission rates of entangled photons.

In this investigation, we have demonstrated both analytically and numerically that a quantum Zeno effect can be employed in birefringent media to suppress PMD. The limitations on the effectiveness of the method are primarily related to how quickly the Zeno effect can be applied before significant PMD affects the photon, the coupling strength itself, and the rate at which the birefringence changes along the length of the waveguide. It should be particularly useful in protecting fragile quantum information encoded in the polarization degree of freedom as it travels through waveguides experiencing significant polarization mode dispersion.

## Data Availability

No new data were created or analyzed in this study. The original contributions presented in this study are included in the article. Further inquiries can be directed to the corresponding author.

## Abbreviations

The following abbreviations are used in this manuscript:

PMD	Polarization Mode Dispersion
PMF	Polarization-Maintaining Fiber

## Appendix A

### Appendix A.1. Time-Dependent Polarization

We assume a single photon enters a birefringent medium, such as an optical fiber, after passing through a narrow-bandwidth filter. The general single-photon state is [28,29]

(A1) $$|\psi \left(0\right)\rangle =c_{N}\int d\omega g\left(\omega \right){\hat{a}}_{k,s}^{†}|0\rangle$$

where g(ω) is approximated by a Gaussian centered on frequency $\omega_{F}$ from the narrow-bandwidth filter. The constant $c_{N}$ is a suitable normalization constant, and the subscripts *k* and *s* refer to the wavenumber and polarizations, respectively. To obtain the time-dependence of the polarization, we apply the electric field operator in one dimension [30]:

(A2) $$\hat{E}\left(x,t\right)=i\sum_{k,s}\sqrt{\frac{2\pi \hbar \omega_{k}}{V}}{\hat{e}}_{k,s}{\hat{a}}_{k,s}e^{i(kx-\omega_{k}t)}.$$

Transforming Equation (A2) into integral form, then applying it to Equation (A1), and further replacing $g\left(\omega \right)\to \exp\left[-{\left(\omega -\omega_{F}\right)}^{2}/\sigma_{F}^{2}\right]$ from the filter, we obtain the time-dependent state

(A3) $$|\psi \left(t\right)\rangle =c_{N}^{\prime }\int d\omega \sqrt{\omega }e^{\frac{-{\left(\omega -\omega_{F}\right)}^{2}}{\sigma_{F}^{2}}}e^{-i\omega t}[e^{ik_{H}x_{H}}|H\rangle -ie^{ik_{V}x_{V}}|V\rangle ].$$

We have assumed that the photon begins in the polarization state $|R\rangle =(|H\rangle -i|V\rangle )/\sqrt{2}$. Noting the narrow bandwidth, we can approximate the slowly varying $\sqrt{\omega }$ as a constant equal to the filter center frequency $\sqrt{\omega_{F}}$. The wavenumber is specified by the polarization state $\hat{e_{k,s}}$ as

(A4) $$k_{s}=k_{0,s}+\alpha_{s}\left(\omega -\omega_{F}\right)+\beta_{s}{\left(\omega -\omega_{F}\right)}^{2}$$

and the frequency under the integral is replaced by

(A5) $$\omega =\omega_{F}+\epsilon .$$

With these replacements, the integral becomes

(A6) $$|\psi \left(t\right)\rangle =c_{N}^{\prime }\int d\epsilon e^{-\frac{\epsilon^{2}}{\sigma_{F}^{2}}}e^{-i\epsilon t}[e^{i\left(\alpha_{H}\epsilon +\beta_{H}\epsilon^{2}\right)x_{H}}|H\rangle -ie^{i\left(\alpha_{V}\epsilon +\beta_{V}\epsilon^{2}\right)x_{V}}|V\rangle ].$$

The factors independent of ϵ have all been absorbed into the normalization constant. Following the identity

(A7) $$\int_{-\infty }^{\infty }dxe^{-(ax^{2}+bx+c)}=\sqrt{\frac{\pi }{a}}e^{(b^{2}-4ac)/4a}$$

we evaluate the integral and obtain

(A8) $$|\psi \left(t\right)\rangle =c_{N}^{\prime \prime }\left(\frac{e^{\frac{{\left(\alpha_{H}x_{H}-t\right)}^{2}}{4(-\frac{1}{2\sigma_{F}^{2}}+i\beta_{H}x_{H})}}}{\sqrt{-\frac{1}{2\sigma_{F}^{2}}+i\beta_{H}x_{H}}}|H\rangle -i\frac{e^{\frac{{\left(\alpha_{V}x_{V}-t\right)}^{2}}{4(-\frac{1}{2\sigma_{F}^{2}}+i\beta_{V}x_{V})}}}{\sqrt{-\frac{1}{2\sigma_{F}^{2}}+i\beta_{V}x_{V}}}|V\rangle \right).$$

Making the replacement $x_{s}\to v_{s}t=ct/n_{s}$, and converting complex numbers into polar form, we obtain

(A9) $$|\psi \left(t\right)\rangle =c_{N}^{\prime \prime }\left(\sqrt{\frac{\gamma_{H}}{\mu_{H}}}e^{\frac{\kappa_{H}^{2}\gamma_{H}t^{2}}{4\mu_{H}}}|H\rangle -i\sqrt{\frac{\gamma_{V}}{\mu_{V}}}e^{\frac{\kappa_{V}^{2}\gamma_{V}t^{2}}{4\mu_{H}}}|V\rangle \right).$$

where

(A10) $$\begin{array}{cc} \kappa_{s} & =(\frac{\alpha_{s}}{n_{s}}c-1),\\ \mu_{s} & =\sqrt{\frac{1}{4\sigma_{F}^{4}}+\frac{\beta_{H}^{2}}{n_{H}^{2}}c^{2}t^{2}},\\ \gamma_{s} & =e^{i\arctan2\sigma_{F}^{2}\frac{\beta_{H}}{n_{H}}ct}. \end{array}$$

Taking t→0, these parameters simplify to $\mu_{s}\to 1/2\sigma_{F}^{2}$ and $\gamma_{s}\to exp\left(2i\sigma_{F}^{2}\beta_{s}^{2}c^{2}t^{2}\right)$. Collecting all the time-dependence in the exponents, and dropping all second-order and higher terms in *t* yield the result $\phi_{s}\approx \sigma_{F}^{2}c\beta_{s}^{2}/n_{s}^{2}$ shown in Equation (8). Taking t→∞ instead simplifies the parameters $\mu_{s}\to \beta_{s}ct/n_{s}$ and $\gamma_{s}\to exp\left(i\pi /2\right)=i$. Canceling a factor of *t* from the numerator and denominator of the exponents gives the alternate result from Equation (8), $\phi_{s}\approx n_{s}/\left(4\beta_{s}c\right){\left(\alpha_{s}/n_{s}-1\right)}^{2}$. Thus, we obtain our approximate form of the normalized polarization time-evolution:

(A11) $$|\psi \left(t\right)\rangle \approx \frac{1}{\sqrt{2}}\left(e^{-i\phi_{H}t}|H\rangle -ie^{-i\phi_{V}t}|V\rangle \right).$$

### Appendix A.2. Quantum Zeno Dynamics

The quantum Zeno effect relies on making frequent measurements to suppress the quantum state evolution in some desirable way [17,18]. We begin by looking at an arbitrary quantum system in a quantum state described by the density matrix ${\hat{\rho }}_{0}$, which evolves according to time-evolution operator $\hat{U}\left(t\right)=exp\left(-i\hat{H}t\right)$, where $\hat{H}$ is a time-independent Hamiltonian. Measurements are denoted by the projection operator $\hat{E}$ into some subspace $H_{E}$ of the total Hilbert space H. ${\hat{\rho }}_{0}$ is assumed to belong to $H_{E}$. The density matrix at some time *T* is found by

(A12) $$\hat{\rho }\left(T\right)=\hat{U}\left(T\right){\hat{\rho }}_{0}{\hat{U}}^{†}\left(T\right).$$

The probability of the quantum state remaining in $H_{E}$ is

(A13) $$P\left(T\right)=Tr[\hat{E}\hat{U}\left(T\right){\hat{\rho }}_{0}{\hat{U}}^{†}\left(T\right)\hat{E}].$$

We note that ${\hat{\rho }}_{0}=\hat{E}{\hat{\rho }}_{0}\hat{E}$. After evolution for time *t* and projection, the matrix is

(A14) $$\begin{array}{cc} \hat{\rho }\left(t\right) & =\hat{E}\hat{U}\left(t\right){\hat{\rho }}_{0}{\hat{U}}^{†}\left(t\right)\hat{E}.\\ \; & =\hat{E}\hat{U}\left(t\right)\hat{E}{\hat{\rho }}_{0}\hat{E}{\hat{U}}^{†}\left(t\right)\hat{E}\\ \; & =\hat{v}\left(t\right){\hat{\rho }}_{0}{\hat{v}}^{†}\left(t\right) \end{array}$$

where $\hat{v}\left(t\right)=\hat{E}\hat{U}\left(t\right)\hat{E}$ is an effective, non-unitary in H time-evolution operator.

Performing *N* measurements over total time *T*, the matrix evolves as

(A15) $${\hat{\rho }}^{\left(N\right)}\left(T\right)={\left[\hat{v}\left(T/N\right)\right]}^{N}{\hat{\rho }}_{0}{\left[{\hat{v}}^{†}\left(T/N\right)\right]}^{N}.$$

If we take the limit N→∞ we can obtain the effective time-evolution operator $\hat{V}\left(T\right)$ for an ideal Zeno effect from continuous measurement.

The system in question involves an incident right-circularly polarized photon, entering a birefringent medium, and the destruction of left-circularly polarized photons serves to project the polarization state back to right-circular. The various terms are, therefore,

(A16) $$\begin{array}{cc} {\hat{\rho }}_{0} & =|R\rangle \langle R|,\\ \hat{E} & =|R\rangle \langle R|,\\ \hat{U}\left(t\right) & =\left[\begin{array}{cc} e^{-i\phi_{H}t} & 0\\ 0 & e^{-i\phi_{V}t} \end{array}\right]. \end{array}$$

Taking $\hat{E}$ and $\hat{U}\left(t\right)$ into matrix form, we evaluate $\hat{v}\left(t\right)$ as

(A17) $$\begin{array}{cc} \hat{v}\left(t\right) & =\hat{E}\hat{U}\left(t\right)\hat{E}\\ \; & =\frac{1}{{\sqrt{2}}^{4}}\left[\begin{array}{cc} 1 & i\\ -i & 1 \end{array}\right]\left[\begin{array}{cc} e^{-i\phi_{H}t} & 0\\ 0 & e^{-i\phi_{V}t} \end{array}\right]\left[\begin{array}{cc} 1 & i\\ -i & 1 \end{array}\right]\\ \; & =\frac{1}{4}\left[\begin{array}{cc} \left(e^{-i\phi_{H}t}+e^{-i\phi_{V}t}\right) & i(e^{-i\phi_{H}t}+e^{-i\phi_{V}t})\\ -i(e^{-i\phi_{H}t}+e^{-i\phi_{V}t}) & \left(e^{-i\phi_{H}t}+e^{-i\phi_{V}t}\right) \end{array}\right]\\ \; & =\frac{1}{4}\left(e^{-i\phi_{H}t}+e^{-i\phi_{V}t}\right)\left[\begin{array}{cc} 1 & i\\ -i & 1 \end{array}\right]\\ \; & =\frac{1}{2}\left(e^{-i\phi_{H}t}+e^{-i\phi_{V}t}\right)|R\rangle \langle R|. \end{array}$$

Taking $\hat{v}\left(t\right)$ to the *N*th power, and replacing t→T/N, we obtain

(A18) $$\begin{array}{cc} {\hat{v}}^{N}\left(\frac{T}{N}\right) & =\frac{1}{2^{N}}{\left(e^{-i\phi_{H}\frac{T}{N}}+e^{-i\phi_{V}\frac{T}{N}}\right)}^{N}(|R\rangle {\langle R|)}^{N}\\ \; & =\frac{1}{2^{N}}{\left(e^{-i\phi_{H}\frac{T}{N}}+e^{-i\phi_{V}\frac{T}{N}}\right)}^{N}|R\rangle \langle R|. \end{array}$$

For a continuous Zeno effect, we will take the limit N→∞. However, we will first transform the complex exponential sum in Equation (A18) into polar form, and distribute the factor of 1/2 into the result.

(A19) $$\begin{array}{cc} \frac{1}{2}\left(e^{-i\phi_{H}\frac{T}{N}}+e^{-i\phi_{V}\frac{T}{N}}\right) & =\frac{1}{2}\sqrt{2+2\cos[\left(\phi_{H}-\phi_{V}\right)\frac{T}{N}]}e^{i\arctan\left[\xi (\frac{T}{N})\right]}\\ \; & =\sqrt{\frac{1+1\cos[\left(\phi_{H}-\phi_{V}\right)\frac{T}{N}]}{2}}e^{i\arctan\left[\xi (\frac{T}{N})\right]}\\ \; & =|\cos\left[\left(\phi_{H}-\phi_{V}\right)\frac{T}{2N}\right]|e^{i\arctan\left[\xi (\frac{T}{N})\right]}. \end{array}$$

Here, we have taken advantage of the identity $\cos\theta^{2}=\left(1+\cos\theta \right)/2$ to simplify the magnitude. The function ξ(T/N) is

(A20) $$\xi \left(\frac{T}{N}\right)=\frac{\sin\left(\phi_{H}\frac{T}{N}\right)+\sin\left(\phi_{V}\frac{T}{N}\right)}{\cos\left(\phi_{H}\frac{T}{N}\right)+\cos\left(\phi_{V}\frac{T}{N}\right)}.$$

We now have the limit

(A21) $$\begin{array}{cc} \lim_{N\to \infty }{\hat{v}}^{N}\left(\frac{T}{N}\right) & =\lim_{N\to \infty }|\cos\left[\left(\phi_{H}-\phi_{V}\right)\frac{T}{2N}\right]{|}^{N}e^{Ni\arctan\left[\xi (\frac{T}{N})\right]}\\ \; & =\lim_{N\to \infty }|\cos\left[\left(\phi_{H}-\phi_{V}\right)\frac{T}{2N}\right]{|}^{N}\lim_{N\to \infty }e^{Ni\arctan\left[\xi (\frac{T}{N})\right]}. \end{array}$$

We can tackle each element of the limit separately, though they will both use similar techniques. In both cases, we will start by setting the argument equal to *y* and taking lny, followed by the use of L’Hopital’s Rule. We start by evaluating the limit of the magnitude factor:

(A22) $$\begin{array}{cc} \lim_{N\to \infty }\ln y & =\lim_{N\to \infty }N\ln\left|\cos\left[\left(\phi_{H}-\phi_{V}\right)\frac{T}{2N}\right]\right|\\ \; & =\lim_{N\to \infty }\frac{\ln|\cos\left[\left(\phi_{H}-\phi_{V}\right)\frac{T}{2N}\right]}{1/N}. \end{array}$$

Applying L’Hopital’s Rule, we obtain

(A23) $$\begin{array}{cc} \lim_{N\to \infty }\ln y & =\lim_{N\to \infty }\frac{\sin\left[\left(\phi_{H}-\phi_{V}\right)\frac{T}{2N}\right]\mathrm{sgn}\left[\cos\left[\left(\phi_{H}-\phi_{V}\right)\frac{T}{2N}\right]\right]\left(\phi_{H}-\phi_{V}\right)\frac{T}{2}\left(-1/N^{2}\right)}{-1/N^{2}|\cos\left[\left(\phi_{H}-\phi_{V}\right)\frac{T}{2N}\right]|}\\ \; & =\lim_{N\to \infty }\frac{\sin\left[\left(\phi_{H}-\phi_{V}\right)\frac{T}{2N}\right]\mathrm{sgn}\left[\cos\left[\left(\phi_{H}-\phi_{V}\right)\frac{T}{2N}\right]\right]\left(\phi_{H}-\phi_{V}\right)\frac{T}{2}}{\left|\cos\left[\left(\phi_{H}-\phi_{V}\right)\frac{T}{2N}\right]\right|}. \end{array}$$

The “sign” function sgn(x) returns 1 if x is positive, −1 if x is negative, and 0 if x is 0. Note that $\lim_{N\to \infty }1/N=\lim_{x\to 0^{+}}x$ since *N* is approaching positive *∞*. The components of the limit are then each evaluated to be

(A24) $$\begin{array}{cc} \; & \lim_{N\to \infty }\sin\left[\left(\phi_{H}-\phi_{V}\right)\frac{T}{2N}\right]=0\\ \; & \lim_{N\to \infty }\mathrm{sgn}\left[\cos\left[\left(\phi_{H}-\phi_{V}\right)\frac{T}{2N}\right]\right]=1\\ \; & \lim_{N\to \infty }|\cos\left[\left(\phi_{H}-\phi_{V}\right)\frac{T}{2N}\right]|=1. \end{array}$$

Thus, the limit is evaluated as

(A25) $$\begin{array}{cc} \; & \lim_{N\to \infty }\ln y=0\\ \Rightarrow & \lim_{N\to \infty }y=1. \end{array}$$

Following a similar process, we evaluate the limit of the complex exponential term. Due to the complexity of the calculation, we will first note the derivative of our term ξ(T/N) with respect to *N*.

(A26) $$\frac{d\xi }{dN}=-\frac{T(\phi_{H}+\phi_{V})}{N^{2}}\frac{1+\cos\left[\left(\phi_{H}-\phi_{V}\right)\frac{T}{N}\right]}{{\left[\cos\left(\phi_{H}\frac{T}{N}\right)+\cos\left(\phi_{V}\frac{T}{N}\right)\right]}^{2}}.$$

Our new limit is

(A27) $$\begin{array}{cc} \lim_{N\to \infty }\ln y & =\lim_{N\to \infty }Ni\arctan\left[\xi \left(\frac{T}{N}\right)\right]\\ \; & =i\lim_{N\to \infty }\frac{\arctan\left[\xi \left(\frac{T}{N}\right)\right]}{1/N} \end{array}$$

and again using L’Hopital’s Rule we obtain

(A28) $$\begin{array}{cc} \lim_{N\to \infty }\ln y & =i\lim_{N\to \infty }\frac{T\left(-1/N^{2}\right)\left(\phi_{H}+\phi_{V}\right)\left[1+\cos\left[\left(\phi_{H}-\phi_{V}\right)\frac{T}{N}\right]\right]}{\left(-1/N^{2}\right)\left[1+\xi^{2}\left(\frac{T}{N}\right)\right]{\left[\cos\left(\phi_{H}\frac{T}{N}\right)+\cos\left(\phi_{V}\frac{T}{N}\right)\right]}^{2}}\\ \; & =i\left(\phi_{H}+\phi_{V}\right)T\lim_{N\to \infty }\frac{\left[1+\cos\left[\left(\phi_{H}-\phi_{V}\right)\frac{T}{N}\right]\right]}{\left[1+\xi^{2}\left(\frac{T}{N}\right)\right]{\left[\cos\left(\phi_{H}\frac{T}{N}\right)+\cos\left(\phi_{V}\frac{T}{N}\right)\right]}^{2}}. \end{array}$$

Looking at the various pieces of the limit, we see that

(A29) $$\begin{array}{cc} \; & \lim_{N\to \infty }\xi \left(\frac{T}{N}\right)=0\\ \; & \lim_{N\to \infty }\cos\left(\phi_{s}\frac{T}{N}\right)=1\\ \; & \lim_{N\to \infty }\cos\left[\left(\phi_{H}-\phi_{V}\right)\frac{T}{N}\right]=1. \end{array}$$

The subscript *s* refers to either *H* and *V*. Therefore, the limit evaluates to

(A30) $$\begin{array}{cc} \; & \lim_{N\to \infty }\ln y=\frac{i}{2}\left(\phi_{H}+\phi_{V}\right)T\\ \Rightarrow & \lim_{N\to \infty }y=e^{\frac{i}{2}\left(\phi_{H}+\phi_{V}\right)T}. \end{array}$$

Thus, the effective time-evolution operator in the limiting case of continuous observation is

(A31) $$\begin{array}{cc} \hat{V}\left(T\right) & \equiv \lim_{N\to \infty }{\hat{v}}^{N}\left(\frac{T}{N}\right)\\ \; & =e^{\frac{i}{2}\left(\phi_{H}+\phi_{V}\right)T}|R\rangle \langle R|. \end{array}$$
